# Associations between Ultrafine Particles and Incident Dementia in Older Adults

**DOI:** 10.1021/acs.est.4c10574

**Published:** 2025-03-13

**Authors:** Qiao Zhu, Yan-Ling Deng, Yang Liu, Kyle Steenland

**Affiliations:** Gangarosa Department of Environmental Health, Rollins School of Public Health, Emory University, Atlanta, Georgia 30322, United States

**Keywords:** air pollution, ultrafine particles, Alzheimer’s disease, dementia, Medicare

## Abstract

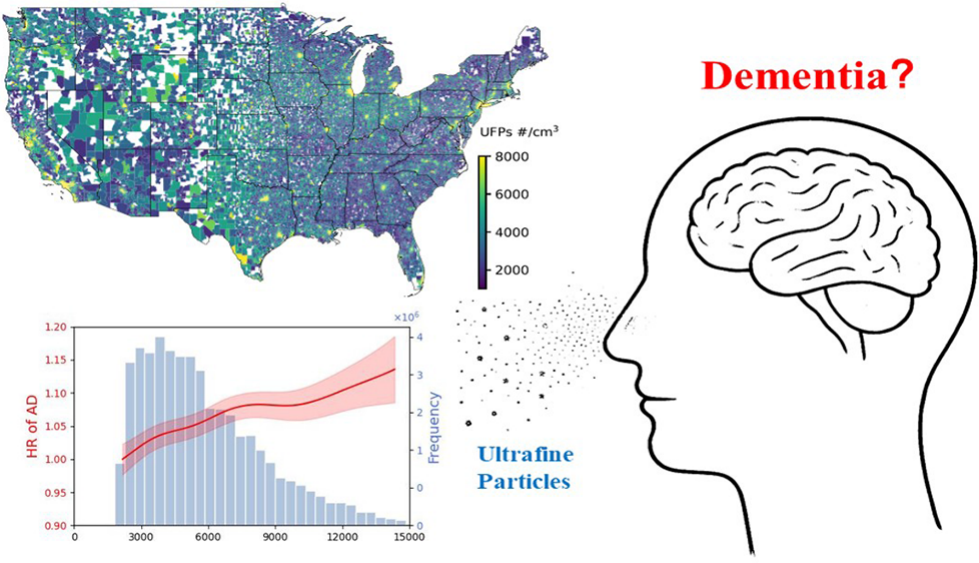

Fine particulate matter (PM_2.5_) is linked to dementia risk, but ultrafine particles (UFPs, <100 nm) may be even more toxic due to their distinct physicochemical properties. However, evidence on UFPs and dementia remains limited. This study assessed the association between UFP exposure and Alzheimer’s disease (AD) and related dementias (ADRD) among U.S. older adults. Using Medicare data, we analyzed ZIP code-level UFP exposure in 2017 for beneficiaries aged 65 and older residing in the contiguous U.S., applying Cox proportional hazard models to estimate AD and ADRD incidence (2018–2020) while considering comorbidities. Among ∼21 million participants for AD and ∼20 million for ADRD, each interquartile range increase in UFP exposure (3701.6 and 3668.5 particles/cm^3^, respectively) was associated with higher AD (HR: 1.026, 95% CI: 1.014–1.038) and ADRD (HR: 1.016, 95% CI: 1.008–1.023) risks. The association was linear within typical exposure levels and stronger in individuals with comorbidities. Geographically, the UFP-associated dementia risk was higher in rural areas than in urban areas, possibly due to different pollution sources. These findings underscore UFPs as neurotoxicants and highlight the need for targeted public health interventions to protect vulnerable populations.

## Abstract 2

This study addresses the lack of research on ultrafine particles, linking long-term exposure to increased dementia risk and highlighting the need for regulatory action.

## 1 Introduction

Dementia (ADRD) mainly affects older adults, and its incidence increases with age. The most common form of dementia is Alzheimer’s disease (AD).^1^ According to the Global Burden of Disease project,^2^ 43.8 million people worldwide had ADRD in 2016, and this number is expected to increase to 152 million by 2050.^3^ This increase makes dementia a major global public health issue, creating significant economic and health burdens for societies and families. Recent studies have reported the impact of fine particulate matter (PM_2.5_) on dementia risk. A review by Wilker et al.^4^ of 14 studies demonstrated a significant increase in dementia risk associated with PM_2.5_ exposure, with a hazard ratio (HR) of 1.04 for every 2 μg/m^3^ increase in PM_2.5_, even at concentrations below the Environmental Protection Agency (EPA)’s annual standard of 12 μg/m^3^. The review also suggested that the risk may plateau at higher concentrations, although evidence on this is limited and variable. Additionally, regional differences in the HR were observed, with Europe showing a particularly elevated ratio of 1.21, compared to 1.03 in North America and 1.04 in Asia, suggesting the global relevance of PM_2.5_’s impact on dementia risk.

Within PM_2.5_, ultrafine particles (UFPs) with a diameter of ≤100 nm present distinct health risks.^5^ Due to their small size, UFPs can easily pass through the blood–brain barrier and invade the central nervous system (CNS) from the lungs. Once in the CNS, UFPs trigger chemical reactions, with their reactive surface and toxic components contributing to pathophysiological processes.^6,7^ Recent animal studies have shown that UFPs in diesel engine emissions significantly affect mouse brains, with male mice exhibiting more severe neuroinflammatory responses than females.^8^ Hassanen et al.^9^ reported that continuous exposure to CuO nanoparticles (a type of UFPs) in rats led to reduced total antioxidant capacity, pathological changes in the cerebrum, hippocampus, and cerebellum, and progressive memory decline. Similarly, studies in Mexico City have linked UFP exposure to early signs of Alzheimer’s, Parkinson’s, and other neurological pathologies, with nanoparticles (diameter ≤ 50 nm) found in critical brain areas of young residents.^10−12^ These findings imply that exposure to UFPs may be associated with a decline in neurocognitive functions.

To the best of our knowledge, epidemiological research on the relationship between ambient UFPs and dementia is scarce. Gan et al.^13^ investigated the impact of UFP exposure on cognitive decline in the elderly in the United States. However, their study did not specifically focus on dementia as an outcome but rather assessed cognitive decline through various functions, such as memory, attention, language, and executive function. Additionally, the study’s relatively small sample size of 5646 participants limits the generalizability of its findings at a national level. Meanwhile, Blanco et al.^14^ studied the link between UFP exposure and dementia cases, but their research was limited to the Seattle area and focused only on traffic-related UFPs, which makes it less applicable to other areas and sources of UFP exposure. To address this knowledge gap, our study investigates the effects of UFP exposure on AD and ADRD in older adults using a large-scale national cohort derived from Medicare data. Additionally, we include PM_2.5_ exposure data using the same method as for UFPs to compare the health effects of UFPs on AD and ADRD with those of PM_2.5_. The initial geographic resolution for UFPs and PM_2.5_ exposure data was at the census block level, which we consolidated to the residential zip code level for our analysis.

## 2 Materials and Methods

### 2.1 Study Population

We utilized two comprehensive, publicly accessible, and privacy-protected databases from the Centers for Medicare and Medicaid Services (CMS): the Medicare Denominator File and the Medicare Chronic Conditions Warehouse (CCW). Using these resources, we established specific cohorts for all-cause Alzheimer’s disease and related disorders (ADRD) and Alzheimer’s disease (AD). Each Medicare beneficiary’s enrollment records, encompassing demographics, Medicaid insurance status (serving as an indicator for socioeconomic status (SES)), date of death (when applicable), and residential zip code, were updated yearly in the denominator file. The CCW claim data encompass predefined indicators for chronic conditions within fee-for-service (FFS) Medicare beneficiaries and furnish the date of the initial diagnosis with a specific condition code. The study participants comprised Medicare beneficiaries who were at least 65 years old, consistently enrolled in the FFS component of Medicare, resided within the contiguous United States, and were covered by both Part A (hospital insurance) and Part B (medical insurance). These inclusion criteria were selected because the CCW relies on FFS, Part A, and Part B for case identification.

UFP data on a national scale were assessed in 2017 and are available only recently (see Section 2.3). Our cohort study was initiated on January 1, 2018, and concluded on December 31, 2020. Following others using Medicare data, we made it more likely that we were studying disease incidence rather than prevalence. We established a minimal clean period of 3 years spanning from 2015 to 2017.^15,16^ The clean period ensured that the subject was free of the outcome of interest for at least three years prior to the first occurrence of the outcome. The inclusion criteria were set with three key conditions: first, the initial diagnosis of the condition under study must occur in 2018 or later, thereby including any cases identified in 2018 itself, as long as they had a 3-year dementia-free clean period in Medicare prior to 2018. Second, entry into the cohort was required to be on or before 2018, ensuring that all participants were included if they joined the cohort at any point up to and including the year 2015. Third, all subjects with the first occurrence of the outcome of interest were required to have joined the cohort at least three years before that first occurrence (clean period). Our research was approved by Emory’s IRB (#RSCH-2020-55733) and the CMS under the data use agreement (#STUDY00000316).

### 2.2 Outcome Classification

In this study, we focused on all-cause ADRD and AD as primary outcomes, aiming to determine the timing of their diagnoses using the CCW algorithm.^17^ AD is a subset of ADRD in the Medicare classification system. The CCW algorithm, validated by studies,^18,19^ uses Medicare claims data—including inpatient, outpatient, and home healthcare claims—to accurately identify individuals diagnosed with these conditions. For the ADRD cohort, the outcome was defined as the first occurrence of either an AD or an ADRD diagnosis code. For the AD cohort, the outcome was defined as either (1) the first occurrence of an AD diagnosis with no prior ADRD diagnosis or (2) the first occurrence of an ADRD diagnosis followed by a subsequent AD diagnosis (assuming that the original ADRD diagnosis was likely AD).

### 2.3 Exposure Assessment

This study used UFP exposure data from Saha et al.,^20^ which estimated UFP concentrations across the continental U.S. using an empirical model. Due to UFPs’ negligible mass concentrations, their concentration is typically represented by particle number concentrations (PNCs). This model used a land-use regression (LUR) framework that combined data from mobile monitoring, fixed site measurements, and key geographic and urban variables. It considered factors such as traffic density, commercial land use, and urbanicity that affect UFP concentrations. By integration of these diverse data sources, the model accurately captured UFP dispersion in urban, suburban, and rural areas. A key feature of this model was its use of high-resolution data to predict PNC for about 6 million residential census blocks, providing annual average UFP concentrations for 2017. The model’s predictions were validated against independent data sets, proving its reliability and accuracy. It showed strong performance with an *R*^2^ value of 0.77 and an RMSE of 2400 particles/cm^3^, along with consistent results from various cross-validation methods, indicating high predictive dependability.

As mentioned earlier, due to extensive research on the effects of PM_2.5_ on dementia, this study focuses on UFPs but also includes PM_2.5_ exposure for comparison. To remain consistent with the method used to predict UFP data, we used the LUR method for PM_2.5_ data as well. Both UFPs and PM_2.5_ data sets can be downloaded from https://www.caces.us/data. Here is a brief overview of the modeling method for PM_2.5_ data.^21^ The model used regulatory monitoring data and approximately 350 geographic characteristics, including traffic, land use, land cover, and satellite-based pollution estimates. It employed universal kriging and partial least-squares (PLS) to summarize geographic variables. Three methods for selecting variables in the PLS model were compared: no variables, a limited number of variables chosen by forward selection, and all variables. The best performance, evaluated using 10-fold cross-validation, came from models using 3–30 selected variables. These models showed a median *R*^2^ of 0.66 with conventional cross-validation and 0.47 with spatially clustered data.

Given the limited availability of long-term UFP monitoring data, Saha et al.^20^ reported that UFP levels showed minimal annual variation (∼2%) and stable spatial patterns across years, especially in urban areas with consistent emission sources. Therefore, our study used 2017 UFP estimates as representative long-term exposure data. In contrast, annual PM_2.5_ estimates from the LUR model were available for multiple years, allowing us to use year-specific PM_2.5_ data to better capture temporal exposure variations. To ensure spatial consistency between exposure estimates and health data, UFP and PM_2.5_ concentrations were aggregated from the census block level to the ZIP code level and then assigned to each subject in the cohort based on their residential ZIP code, accounting for any annual residential mobility changes as recorded in the Medicare database. The ZIP codes in our study have a mean area of 236.66 km^2^ (SD = 438.96 km^2^), ranging from 0.031 to 4984 km^2^.

### 2.4 Covariates

To ensure a comprehensive selection of confounders and to illustrate the assumed relationships among UFP exposure, covariates, and the outcome (incident dementia), we constructed a directed acyclic graph (Figure S1) based on previous studies.^15,16^ In our study, we obtained individual-level data such as sex, race, age at entry, and Medicaid eligibility from the Medicare denominator file. We also included various neighborhood-level factors associated with air pollution and cerebrovascular diseases. These factors at the zip code level included SES indicators such as population density, the proportion of elderly living below the poverty line, racial demographics, household income, homeowner rates, and education levels among the elderly. Environmental and health-related variables were also considered, including annual average temperature and humidity and county-level metrics on lifestyle risk factors (average body mass index and smoking rates) and healthcare resources (hospital and doctor availability) across five U.S. regions (West, Southwest, Midwest, Northeast, and Southeast).

Our data sources included the 2000^22^ and 2010 U.S. Census,^23^ the American Community Survey (2005–2012),^24^ meteorological data from the North American Regional Reanalysis (NARR) for 2000–2017,^25^ the Behavioral Risk Factor Surveillance System for behavioral risk factors (2000–2016),^26^ and the American Hospital Association for healthcare capacity (2010, 2015, 2018).^27^ Missing data were addressed through linear interpolation or extrapolation. Based on findings from Steenland et al.,^28^ we identified stroke, hypertension, and depression as key predictors of AD and ADRD from the CCW databases. This study aims to investigate the potential modification effects of these comorbidities on the association between UFPs and AD/ADRD.

### 2.5 Statistical Analysis

We used stratified Cox proportional hazards models and a generalized estimating equation (GEE) approach to assess the impact of UFPs and PM_2.5_ exposure on the risk of AD and ADRD in the elderly. Stratified Cox models were chosen for their robustness in handling time-to-event data, while the GEE approach accounted for clustering within ZIP codes, providing valid inference through robust standard errors.^29^ These models controlled for residual autocorrelation within zip codes using robust standard errors and estimated HRs and 95% confidence intervals (CIs) for each pollutant per interquartile range (IQR) increase in mean concentrations. We included the covariates based on the directed acyclic graph constructed to illustrate the relationships among UFP exposure, covariates, and incident dementia, guided by previous studies.^15,16^ We ran single-pollutant models for UFPs and PM_2.5_ to evaluate each effect on the health outcomes. Stratification was based on Medicaid insurance status, single-year age at entry, sex, and race, with adjustments for neighborhood-level SES, behavioral risk factors, and healthcare capacity. The models also included a linear term for calendar years and geographical region indicators to account for residual temporal and spatial trends.

To assess the concentration–response (C–R) relationship between UFPs and PM_2.5_ and AD or ADRD, we used penalized splines for each pollutant while adjusting for covariates. We examined potential effect modifiers by incorporating interaction terms between pollutants and age (<75 vs ≥75 years), sex, race (White, Black, and Other), Medicaid eligibility (ineligible vs ever eligible), five regions (i.e., Midwest, Northeast, Southeast, Southwest, and West, as shown in Figure S2), urbanization status, and comorbidities (stroke, hypertension, and depression). Urbanization status was classified based on the 2010 Rural-Urban Commuting Area (RUCA) Codes, with RUCA codes 1–9 designated as urban and code 10 as rural.

To ensure the reliability of our results, we conducted several sensitivity analyses. First, we developed bipollutant models that included both UFPs and PM_2.5_. Second, we established a 5-year “clean period,” similar to the 3-year “clean period” described in Section 2.1, excluding participants diagnosed with AD or ADRD within the first five years of follow-up to minimize the potential for reverse causation. Third, to address potential bias from residential mobility, we conducted a sensitivity analysis limited to participants who remained in the same location throughout the follow-up period (i.e., the nonmover cohort). Then, to further assess the robustness of these associations against unmeasured confounding, we calculated E-values for our main exposure-outcome associations (UFPs and PM_2.5_ with ADRD/AD), using Quartile 1 vs Quartile 4 as the reference and exposed groups. The *E*-value quantifies the minimum strength of association that an unmeasured confounder would need to have with both the exposure and the outcome to fully explain the observed association, assuming no other confounding. Using the formula from VanderWeele and Ding:^30^
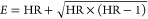
1

Finally, a sensitivity analysis compared HRs before and after adjusting for stroke, hypertension, and depression to evaluate whether these comorbidities act as confounders or intermediates in the causal pathway. All analyses were performed on the Rollins HPC Cluster at Emory University using R software version 4.2.3, with a significance threshold of *P* < 0.05.

## 3 Results

### 3.1 Study Population Characteristics

**Figure 1:**
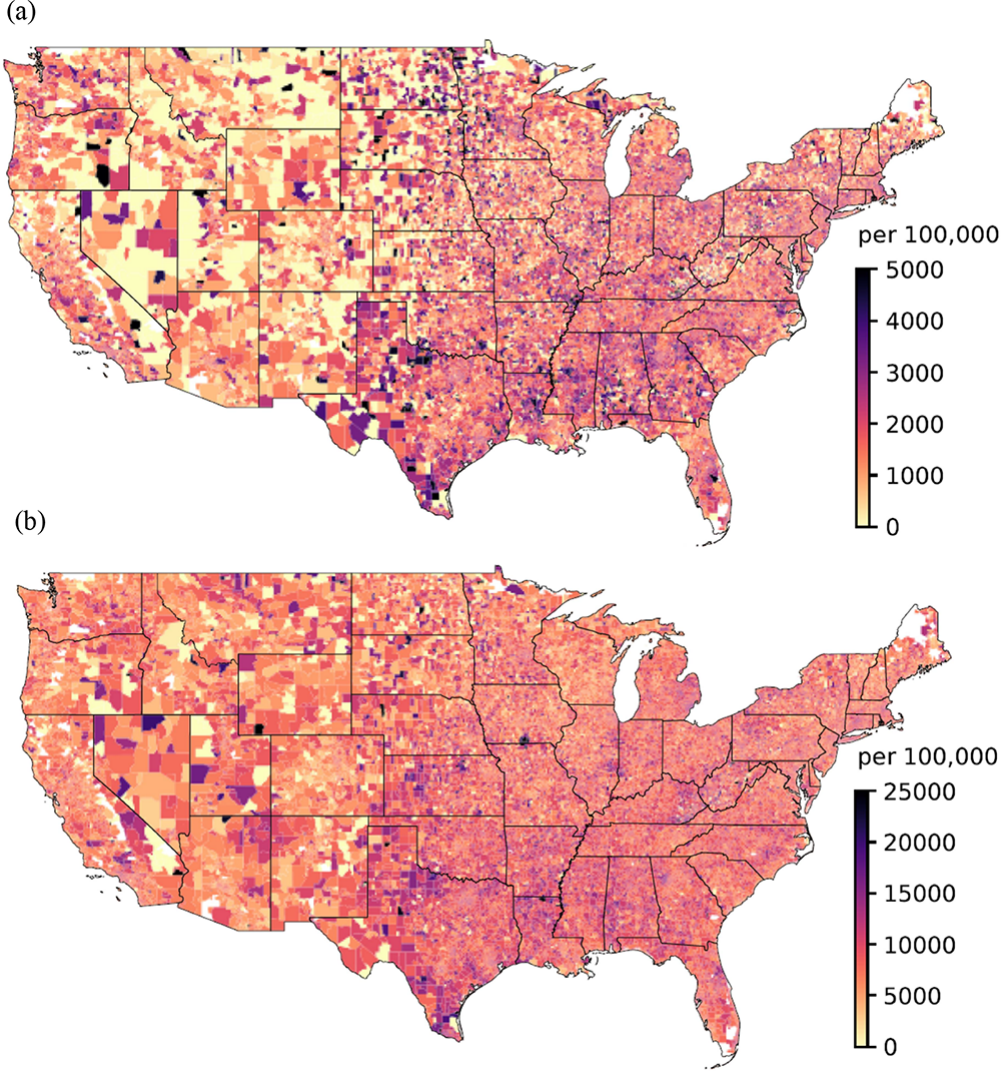
Prevalence of initial AD (a) and initial ADRD (b) incidents per 100,000 Medicare beneficiaries throughout the contiguous United States from 2018 to 2020, considering a 3-year clean period.

Table 1 presents key statistics for individuals with AD and ADRD in our study cohorts from 2018 to 2020, consisting of 20,763,472 and 19,255,995 participants, respectively. In the AD cohort, there were 380,675 events (1.8% of the population), whereas the ADRD group saw 1,713,541 events (8.9%). In both cohorts, the proportion of participants aged 75 and older was slightly higher, accounting for 52.3% in the AD group and 50.2% in the ADRD group. For the gender distribution, females make up a higher proportion than males, accounting for 56.0% in the AD group and 55.6% in the ADRD group. The racial demographics show a predominantly white majority, exceeding 87% in both groups. Medicaid eligibility reveals a high proportion of nondual eligible participants (who were not on Medicaid), at 89.0% in AD and 90.4% in ADRD. The prevalence of comorbidities, such as stroke, hypertension, and depression, was notable across both cohorts, with hypertension affecting over 81% and depression around 32–34% in each cohort. Stroke was less common, impacting approximately 15% of the individuals. Only 14–15% had no comorbidities in either cohort. Air pollution exposure, assessed by PM_2.5_ and UFP levels, was comparable between the two cohorts. The median values of PM_2.5_ were 7.1 μg/m^3^, while UFP particle counts were 5213.2 particles/cm^3^ for the AD cohort and 5190.6 particles/cm^3^ for the ADRD cohort. Occurrences of first ADRD and AD events per 100,000 Medicare beneficiaries at the Zip code level across the contiguous United States are presented in Figure 1.

**Table 1:** Descriptive Statistics for the Study Population and Distribution of Air Pollution after a 3-Year Clean Period [Mean (SD) or *n* (%)]

variables	AD	ADRD
Characteristics		
number of events	380,675 (1.8)	1,713,541 (8.9)
number of the total population	20,763,472 (100.0)	19,255,995 (100.0)
total person-years	54,062,424 (100.0)	49,463,902 (100.0)
median follow-up years	3	3
**Age at entry (years)**	76.60 (7.08)	76.14 (6.77)
<75	9,899,617 (47.7)	9,596,323 (49.8)
≥75	10,863,855 (52.3)	9,659,672 (50.2)
Sex		
male	9,128,993 (44.0)	8,542,541 (44.4)
female	11,634,479 (56.0)	10,713,454 (55.6)
Race		
white	18,051,472 (86.9)	16,762,914 (87.1)
black	1,358,666 (6.5)	1,231,018 (6.4)
other^a^	1,353,334 (6.5)	1,262,063 (6.6)
Medicaid eligibility		
ineligible	18,469,536 (89.0)	17,409,569 (90.4)
ever eligible	2,293,936 (11.0)	1,846,426 (9.6)
Comorbidities		
stroke	3,416,393 (16.5)	2,799,888 (14.5)
hypertension	17,116,380 (82.4)	15,676,493 (81.4)
depression	7,162,439 (34.5)	6,171,944 (32.1)
no comorbidities^b^	2,861,443 (13.8)	2,833,034 (14.7)
Air pollutants^c^		
PM_2.5_ (μg/m^3^)	7.1 (1.9)	7.1 (1.9)
UFPs (particles/cm^3^)	5213.2 (3701.6)	5190.6 (3668.5)

a Other included Asian, Hispanic, American Indian, or Alaskan Native, and unknown.

b Means none of the above comorbidities.

c Presented as median concentration (IQR).

### 3.2 Air Pollution Levels

**Figure 2:**
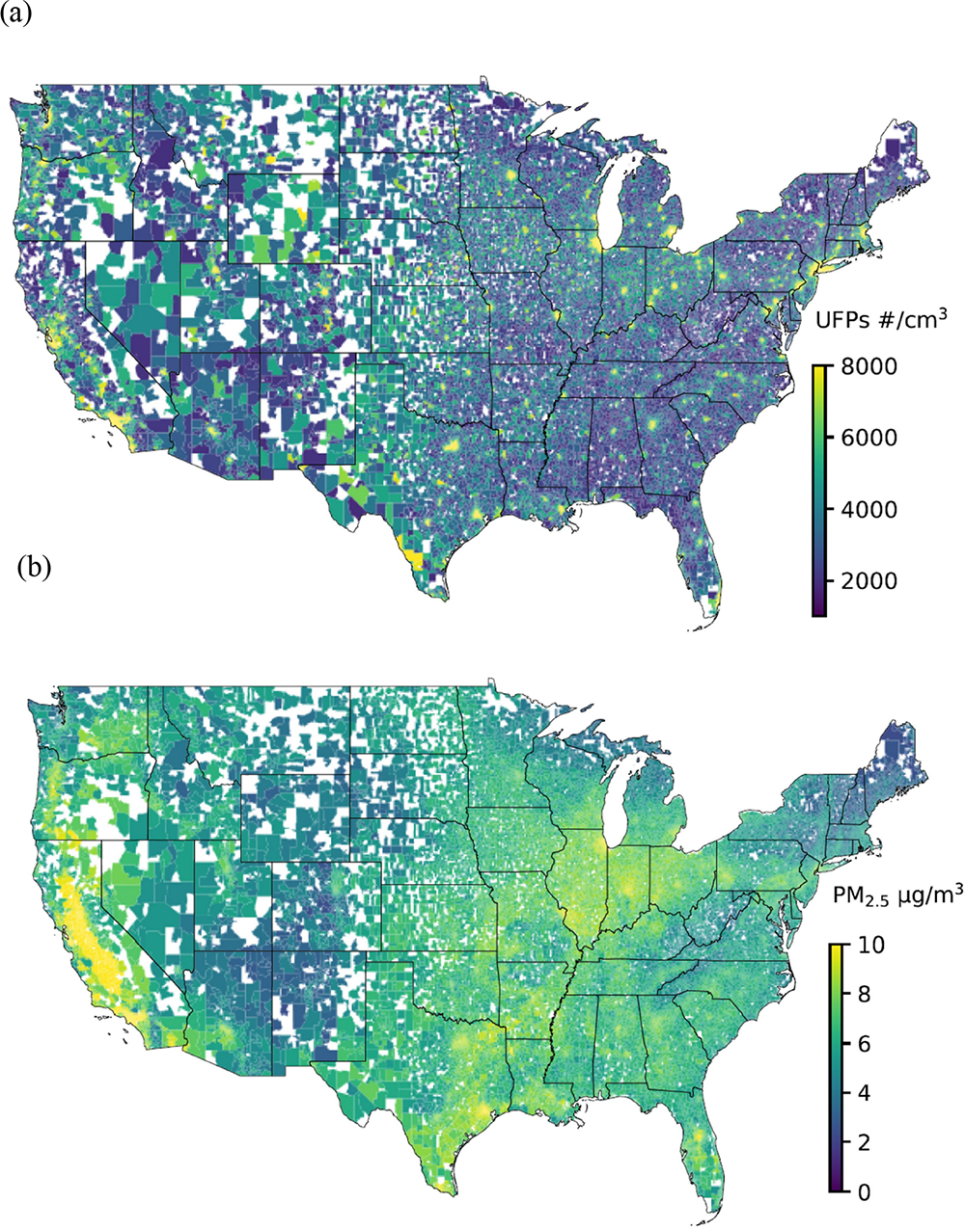
Average concentrations of (a) UFPs (particles/cm^3^) and (b) PM_2.5_ (μg/m^3^) across the contiguous United States based on the exposure data used in this study. Blank areas represent locations where missing data prevented model predictions.

During the study period, the average annual PM_2.5_ concentration among participants in both the AD and ADRD cohorts was 7.2 μg/m^3^, ranging from 1.3 to 33.37 μg/m^3^, consistently staying below the 12 μg/m^3^ annual standard established by the United States EPA. However, this level exceeded the World Health Organization’s (WHO) most recently established annual average guideline for PM_2.5_, which is 5 μg/m^3^. For UFPs, the annual mean concentration was 5792.3 particles/cm^3^, ranging from 1807.4 to 19,784 particles/cm^3^ in the AD cohort and 5765.6 particles/cm^3^ (ranging from 1807.4 to 19,784 particles/cm^3^) in the ADRD cohort. It is important to note that currently no ambient air-quality standards have been established for UFPs. The Spearman correlation coefficient for the concentrations of UFPs and PM_2.5_ across zip codes is 0.45, indicating significant differences in their spatial distribution patterns. High concentrations of UFPs are primarily found in urban areas with dense transportation networks. In contrast, high PM_2.5_ concentrations display a more widespread pattern, particularly elevated in California and Northwestern regions (Figure 2).

### 3.3 Health Effect Estimates

Table 2 displays the results from single-pollutant Cox proportional hazards models, adjusted for individual characteristics, neighborhood-level SES, behavioral risk factors, and healthcare capacity variables while also accounting for residual temporal and spatial trends. This table illustrates the association between exposure to both UFPs and PM_2.5_ and the risk of developing AD and ADRD. For UFPs, each IQR increase is associated with an HR of 1.026 (95% CI: 1.014, 1.038) for AD and 1.016 (95% CI: 1.008, 1.023) for ADRD, with significant trends observed across quartiles. For PM_2.5_, each IQR increase corresponds to an HR of 1.037 (95% CI: 1.030, 1.045) for AD and 1.017 (95% CI: 1.012, 1.021) for ADRD, with similar significant trends across quartiles. These findings suggest that higher exposure levels to UFPs and PM_2.5_ are both significantly associated with increased risks of both AD and ADRD. The IQRs for UFPs are 3701.6 particles/cm^3^ in the AD cohort and 3668.5 particles/cm^3^ in the ADRD cohort, while the IQR for PM_2.5_ is 1.9 μg/m^3^ in both cohorts.

**Table 2:** HRs and 95% CIs of UFPs and PM_2.5_ Associated with AD or ADRD

exposure	AD	ADRD
UFPs (particles/cm^3^)		
continuous^a^	1.026 (1.014, 1.038)	1.016 (1.008, 1.023)
Quartile 1	reference	reference
Quartile 2	1.034 (1.016, 1.051)	1.036 (1.026, 1.046)
Quartile 3	1.042 (1.023, 1.061)	1.042 (1.030, 1.053)
Quartile 4	1.048 (1.026, 1.070)	1.041 (1.027, 1.054)
PM_2.5_ (μg/m^3^)		
continuous^a^	1.037 (1.030, 1.045)	1.017 (1.012, 1.021)
Quartile 1	reference	reference
Quartile 2	1.051 (1.036, 1.067)	1.042 (1.034, 1.051)
Quartile 3	1.079 (1.062, 1.096)	1.062 (1.052, 1.072)
Quartile 4	1.099 (1.080, 1.119)	1.064 (1.052, 1.075)

a Results are presented in the unit of per IQR change of exposure.

**Figure 3:**
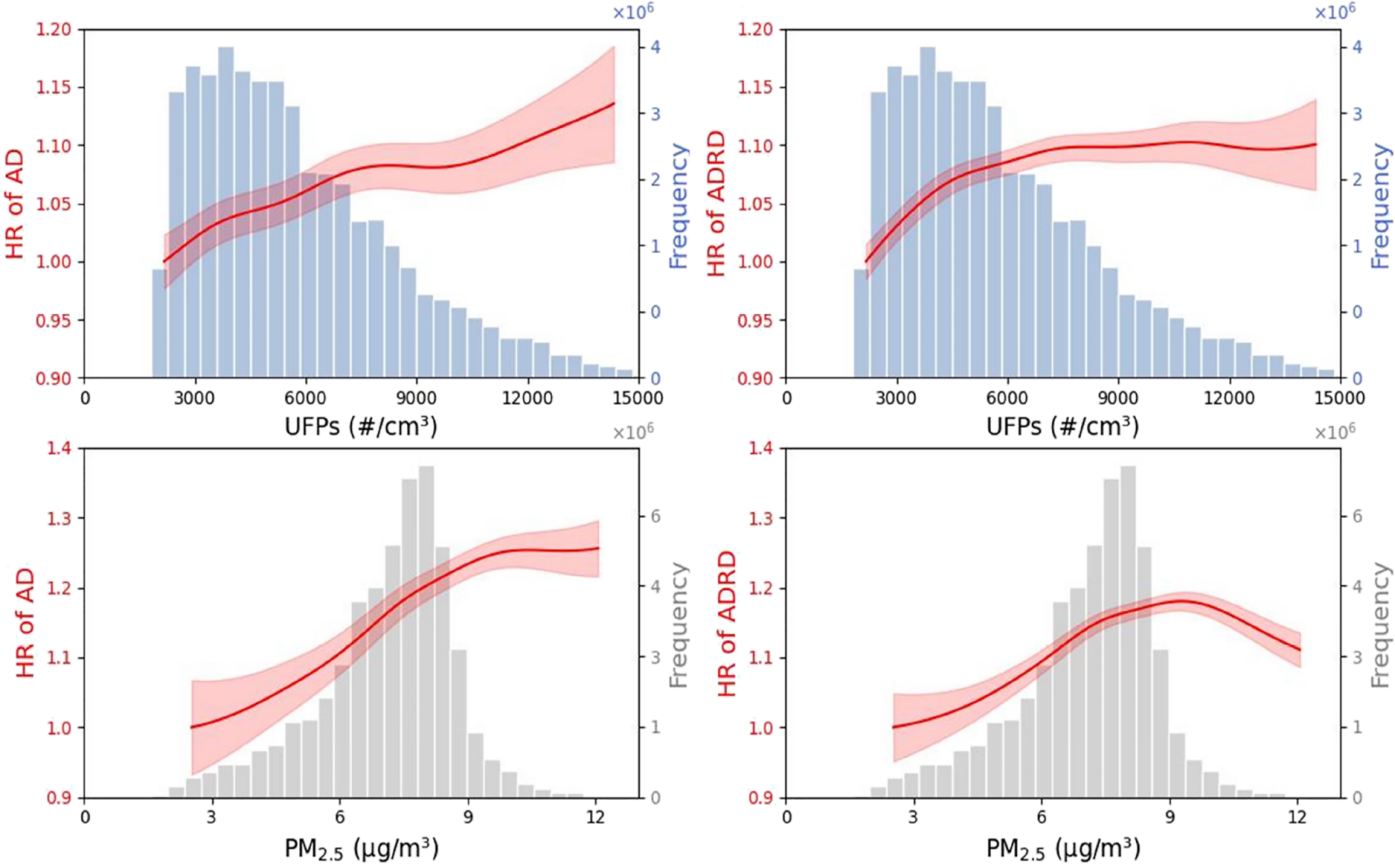
C-R curves for UFPs and PM_2.5_ in relation to AD and ADRD, based on single-pollutant models, displayed over the 0.05th to 99.95th percentile range of concentrations.

Figure 3 shows the penalized spline curves for UFPs and PM_2.5_ from single-pollutant models. For UFPs, within the most observed concentration range (around 3000–8000 particles/cm^3^), the C-R curves for both AD and ADRD display a predominantly linear pattern. Beyond 8000 particles/cm^3^, the C-R curve enters a brief plateau phase before continuing with a linear increase. The C-R relationships for PM_2.5_ show a generally linear upward trend for AD, and for ADRD, a clear linear increase is also evident at concentrations below 9 μg/m^3^. Overall, for both UFPs and PM_2.5_, within their most frequently observed concentration ranges, the impacts on AD and ADRD become increasingly pronounced as the concentration increases.

### 3.4 Effect Modifications

We observed varying strengths in the associations among UFPs, PM_2.5_, and the risk of AD and ADRD in single-pollutant models across different subgroups, depending on the presence or absence of specific comorbidities (Table 3). Generally, the associations among UFPs, PM_2.5_, and the risk of AD and ADRD were stronger and more evident in individuals with comorbidities. Particularly for UFPs, all HRs were positive in individuals with comorbidities for both the AD and ADRD groups and were higher than those without comorbidities of the same type, especially in individuals with depression. These associations emphasize the need to consider comorbid health conditions when assessing the impact of UFPs on the risk of neurodegenerative diseases.

**Table 3:** Subgroup Analysis by Comorbidities of HRs and 95% CIs per IQR Increase in UFPs and PM_2.5_ Associated with AD or ADRD^a^

	UFPs		PM_2.5_	
subgroup	HR (95% CI)	*P*-value^b^	HR (95% CI)	*P*-value^b^
AD				
with stroke	1.032 (1.015, 1.050)	0.334	1.045 (1.035, 1.056)	0.035
without stroke	1.022 (1.009, 1.035)		1.031 (1.023, 1.039)	
with hypertension	1.027 (1.014, 1.039)	0.212	1.035 (1.028, 1.043)	0.855
without hypertension	1.006 (0.976, 1.036)		1.033 (1.014, 1.053)	
with depression	1.035 (1.021, 1.050)	0.005	1.044 (1.036, 1.053)	0.019
without depression	1.005 (0.990, 1.021)		1.029 (1.019, 1.038)	
ADRD				
with stroke	1.019 (1.011, 1.028)	0.303	1.011 (1.007, 1.016)	0.633
without Stroke	1.013 (1.005, 1.021)		1.013 (1.008, 1.018)	
with hypertension	1.016 (1.008, 1.023)	0.008	1.011 (1.007, 1.016)	0.435
without hypertension	0.991 (0.974, 1.008)		1.016 (1.006, 1.026)	
with depression	1.028 (1.020, 1.036)	<0.001	1.021 (1.017, 1.026)	0.007
without depression	0.994 (0.984, 1.003)		1.011 (1.006, 1.017)	

a All results were presented in the unit of per IQR change of exposure. In the AD cohort, the IQRs for UFPs and PM_2.5_ were 3701.6 particles/cm^3^ and 1.9 μg/m^3^, respectively. In the ADRD cohort, the IQRs for UFPs and PM_2.5_ were 3668.5 particles/cm^3^ and 1.9 μg/m^3^, respectively.

b *P*-value for the interaction term was estimated by the Wald test.

We also explored several potential effect modifiers (Table S2). For UFPs, the effects on AD and ADRD were more pronounced in individuals aged 75 years or older, whereas for PM_2.5_, the effects were more significant in those under 75 years. Among the sex subgroups, the impacts of both UFPs and PM_2.5_ on AD and ADRD were greater in males. Furthermore, the influence of UFPs on AD and ADRD was more pronounced in individuals who were eligible for Medicaid. The associations between UFPs and PM_2.5_ with the risk of AD and ADRD varied across different racial groups. UFPs showed positive associations with AD in White and “Other” groups, but not in Black individuals, and were positively associated with ADRD only in White individuals. In contrast, PM_2.5_ was positively associated with both AD and ADRD in White and Black groups, with the strongest effects for AD in Black individuals and for ADRD in White individuals. We observed substantial geographic variations in the associations among UFPs, PM_2.5_, and the risk of AD and ADRD. The strongest associations for both pollutants and outcomes were observed in the Midwest, where the HRs for UFPs reached 1.127 (95% CI: 1.097–1.158) for AD and 1.075 (95% CI: 1.054–1.096) for ADRD. In the Northeast, UFP exposure had a positive association with AD and ADRD, while PM_2.5_ showed minimal effects. The Southeast showed stronger associations for PM_2.5_ but weaker effects for UFPs. In the Southwest, UFP exposure had a notable effect on AD, while PM_2.5_ was more strongly associated with ADRD. In the West, UFP exposure had positive associations with both AD and ADRD. For urbanization, both UFP and PM_2.5_ exposures showed stronger associations in rural areas (UFP: HR = 1.185 for AD and 1.122 for ADRD; PM_2.5_: HR = 1.085 for AD and 1.045 for ADRD) compared to urban areas (UFP: HR = 1.012 for AD and 1.004 for ADRD; PM_2.5_: HR = 1.061 for AD and 1.033 for ADRD).

### 3.5 Sensitivity Analyses

The associations between exposure to UFPs and PM_2.5_ and the risk of AD and ADRD remained consistent across multiple sensitivity analyses.

First, in the bipollutant models (Table S3), HRs for both UFPs and PM_2.5_ were slightly lower than in single-pollutant models but remained comparable, indicating robust associations with AD and ADRD. A significant interaction (*p* < 0.001) suggested a synergistic effect between the two pollutants. Second, we refined the cohorts by excluding participants diagnosed with AD or ADRD within the first 5 years of follow-up to address potential reverse causation. The results from these adjusted cohorts were consistent with the primary analyses based on 3 years (Table S4), suggesting that early diagnoses did not significantly bias our findings. Third, restricting the analysis to nonmovers (Table S5) produced HRs similar to those of the main analysis, reinforcing the robustness of our findings despite potential exposure misclassification due to residential mobility.

Then, based on our E-value calculations, we found that for UFP exposure the observed HR for AD was 1.048, corresponding to an E-value of 1.30, while for ADRD, the HR was 1.041, with an E-value of 1.27. For PM_2.5_ exposure, the HR for AD was 1.099, yielding an E-value of 1.50, whereas for ADRD, the HR was 1.064, corresponding to an E-value of 1.33. These values indicate that an unmeasured confounder would need to have a minimum association of 1.27–1.50 with both UFP/PM_2.5_ exposure and AD/ADRD risk to fully account for the observed relationships. However, the positive exposure–response relationship, which monotonically increases across quartiles for both UFP and PM_2.5_ in AD/ADRD, provides further evidence against the presence of a single unmeasured confounder driving the associations, as such a confounder would also need to be associated with both exposure and outcomes in a similar monotonic confounder-response trend. The main known potential confounder not controlled for in our work is the APOE genetic variation, a well-known established genetic risk factor for AD/ADRD.^31^ However, there is no a priori reason to expect that the APOE status would be systematically associated with exposure to UFPs or PM_2.5_. Without such an association, APOE cannot be a confounder.

Last, Table S6 shows that adjusting for stroke, hypertension, and depression resulted in minimal changes in HRs, suggesting that these comorbidities are unlikely to be confounders or mediators in the associations among UFPs, PM_2.5_, and dementia risk. Instead, our stratified analyses (Table 3) and interaction tests indicate that these comorbidities may act as effect modifiers, with stronger effects of both pollutants observed in individuals with these comorbidities.

## 4 Discussion

This national longitudinal study included a large U.S. cohort of about 21 million participants for AD and 20 million participants for ADRD from 2018 to 2020. We observed a notable increase in HRs for both AD and ADRD in association with both UFPs and PM_2.5_. The associations were stronger for PM_2.5_ than UFPs, suggesting that there are other components of PM_2.5_ that add further risk on top of UFPs, but that UFPs do play an important role. The C-R curves from our models show that the HRs for AD and ADRD generally follow a linear upward trend across most concentration ranges of UFPs and PM_2.5_. Additionally, our subgroup analyses indicated that the effects of UFPs on AD and ADRD were stronger in individuals with comorbidities such as stroke, hypertension, and depression, suggesting increased vulnerability due to underlying health conditions. The impact of UFPs was also greater in those aged 75 and older, while PM_2.5_ had a stronger effect on individuals under 75. Both UFPs and PM_2.5_ had more pronounced effects on males and those eligible for Medicaid, highlighting socioeconomic disparities. There were also racial differences: UFPs were linked to higher AD risk in White and “Other” groups but not in Black individuals, while PM_2.5_ was linked to higher AD and ADRD risk in White and Black groups. UFP associations with dementia risk varied geographically, with the strongest effects in the Midwest and positive associations in the Northeast, Southwest, and West. Effects were more pronounced in rural areas than in urban areas, potentially due to differences in pollution sources and dispersion patterns. Our findings align with recent research reporting no strong association between traffic-related UFP exposure and dementia risk.^14^ This consistency suggests that urban UFP exposure, primarily driven by traffic emissions, may have a weaker impact compared with UFP sources prevalent in rural areas, such as biomass burning and wildfires, which are significant contributors to UFP concentrations.

Our study findings are consistent with recent studies showing that PM_2.5_ has an impact on dementia.^4^ Regarding the relationship between UFPs and dementia or cognitive decline, current epidemiological studies, while showing no significant association between UFP exposure and these outcomes, are limited by small sample sizes and restricted study areas.^13,14^ However, more evidence from neurotoxicology studies is emerging that demonstrates a strong link between UFP exposure and neurodegenerative processes.^10−12^ For example, Calderón-Garcidueñas et al.^32^ provide pathological evidence that nanoparticles, especially combustion-derived nanoparticles, are linked to early and progressive damage to the neurovascular unit, accumulation of hyperphosphorylated tau and amyloid-beta, oxidative stress, mitochondrial dysfunction, and neuroinflammation, all of which are recognized contributors to neurodegenerative diseases like AD.^11^ This indicates that despite the current limitations of epidemiological studies, UFPs may have a substantial impact on the pathogenesis of dementia. Given the very limited literature in this area, more research is warranted to better understand these potential effects.

Our study has several strengths. First, it is a comprehensive national cohort analysis of the impact of UFPs on AD and ADRD in the U.S., which has the statistical power to detect subtle effects common to environmental health studies. Second, by using Medicare claims data, which includes both physician visits and hospital admissions, we broaden case identification, capturing cases diagnosed in nonhospital settings or at early stages, leading to a more accurate estimate of incidence rates compared to studies relying only on hospitalization data. Third, our analysis incorporates a 3-year “clean” period and focuses on participants with continuous enrollment in Medicare Fee for Service, Part A, and Part B programs, ensuring the inclusion of only newly diagnosed cases and providing a more precise estimate of incidence. Additionally, our control for a wide range of covariates at both the individual and neighborhood levels enhances the reliability of our findings.

Despite the strengths of our study, certain limitations must be acknowledged. First, our estimation of UFP exposure was based on a nationwide LUR model rather than direct measurements; this model accounted for 77% of the spatial variation in UFP exposure, as reported by Saha et al.^20^ While the model provides high-resolution predictions at the census block level, we aggregated these estimates to the ZIP code level to align with Medicare data. Although this aggregation smoothes fine-scale spatial variability, prior studies show that ZIP code-level estimates can still capture meaningful air pollution–AD associations.^15,16^ Although UFP concentrations at the ZIP code level in our study appear narrower than those modeled at the block level by Saha et al.,^20^ the spatial distribution of high UFP values remains consistent across regions, aligning with our focus on large-scale exposure–response relationships. We recognize that spatial aggregation may introduce nondifferential exposure misclassification. However, similar to PM_2.5_ assignment at the ZIP code level, this is likely a Berkson error, which does not introduce systematic bias but may slightly reduce precision.^33^ Moreover, our study examines UFP number concentrations without detailed size distribution data, which may influence the observed associations. Recent findings^34^ suggest that UFP size fractions could confound these relationships, highlighting the need for further investigation. Additionally, there is a risk of outcome misclassification due to our reliance on administrative records for case identification. Specifically, AD cases constitute only 22% of all dementia diagnoses in our data set, which could suggest potential underdiagnosis, as AD usually represents approximately 2/3rd of the ADRD cases.^16^ However, the previous work^16^ had demonstrated that such misclassification is unlikely to have a marked impact on our results, as detailed in the Supporting Information. Another limitation is related to the confounder adjustment, which was restricted to factors inferable from neighborhood-level data.

In conclusion, our national longitudinal analysis demonstrates a significant association between UFP exposure and an increased risk of developing AD and ADRD in a cohort of over 41 million U.S. residents from 2018 to 2020. The results show that higher UFP levels are linked to greater risks of AD and ADRD, with a consistent trend observed across the data. The risk associated with exposure to UFPs increases markedly with rising concentrations within common exposure ranges. Furthermore, the study indicates that individuals with comorbidities, such as stroke, hypertension, and depression, are at a higher risk of adverse effects from UFP exposure on neurodegenerative diseases. These findings suggest the need for public health measures and policies to reduce UFP exposure, particularly among vulnerable populations, to decrease the incidence of AD and ADRD.

## Supporting Information Available

Details regarding the distribution of UFPs and PM_2.5_ levels over the study period; subgroup analyses of HRs and CIs across key demographic and geographic characteristics; single- and bipollutant model comparisons for AD and related dementias (ADRD); sensitivity analyses incorporating a five-year clean period and a nonmover cohort; HR estimates adjusted for comorbidities; directed acyclic graph illustrating the hypothesized relationships among UFPs, PM_2.5_, and dementia risk; and a geographical map depicting the regional study areas within the contiguous United States (PDF)
