# Supplementary material for: Associations between Ultrafine Particles and Incident Dementia in Older Adults
Source: Environ Sci Technol. 2025 Mar 13;59(11):5443–51. doi: 10.1021/acs.est.4c10574 (PMC11948469; doi:10.1021/acs.est.4c10574)
Supplement: Supplementary file 1 — es4c10574_si_001.pdf [file es4c10574_si_001.pdf]

Supplemental Material

Associations between Ultrafine particles with Incident Dementia in Older adults

Qiao Zhu [a,1], Yan-Ling Deng [a,1], Yang Liu [a,*], Kyle Steenland [a]

[a]*Gangarosa Department of Environmental Health, Rollins School of Public Health, Emory University, Atlanta, GA 30322, USA*

[1]*These authors contributed equally*

* Email: yang.liu@emory.edu

This file contains 7 pages, including 6 tables and 2 figures.

Table S1. Distributions of annual UFPs and $PM_{2.5}$ levels over the study period.

Exposure	Min	5%	25%	50%	75%	95%	Max	Mean
**AD cohort**								
UFPs (particles/cm^3)	1807.39	2430.33	3663.22	5213.24	7364.79	11275.8	19784	5792.27
$PM_{2.5}$ (μg/m^3)	1.30	4.70	6.12	7.06	8.02	10.02	33.37	7.19
**ADRD cohort**								
UFPs (particles/cm^3)	1807.39	2423.91	3654.6	5190.62	7323.1	11214.7	19784	5765.61
$PM_{2.5}$ (μg/m^3)	1.30	4.69	6.11	7.05	8.02	10.00	33.37	7.18

Table S2. Subgroup analysis by characteristics of hazard ratios and 95% CIs of per IQR increase in UFPs and $PM_{2.5}$ associated with AD or ADRD.

Subgroup	AD				Dementia			
	UFPs		$PM_{2.5}$		UFPs		$PM_{2.5}$	
	HR (95% CI)	P value[a]	HR (95% CI)	P value[a]	HR (95% CI)	P value[a]	HR (95% CI)	P value[a]
Age at entry (years)								
<75	1.036 (1.014, 1.059)	0.039	1.051 (1.037, 1.065)	0.253	1.051 (1.038, 1.064)	0.271	1.027 (1.020, 1.035)	0.326
≥75	1.064 (1.051, 1.078)		1.042 (1.034, 1.049)		1.060 (1.051, 1.068)		1.023 (1.018, 1.027)	
Sex								
Male	1.027 (1.012, 1.043)	0.857	1.039 (1.03, 1.049)	0.669	1.027 (1.018, 1.035)	0.002	1.017 (1.011, 1.022)	0.997
Female	1.025 (1.012, 1.039)		1.036 (1.028, 1.045)		1.008 (1.000, 1.017)		1.017 (1.012, 1.021)	
Race								
White	1.027 (1.014, 1.039)	Reference	1.039 (1.031, 1.047)	Reference	1.017 (1.009, 1.025)	Reference	1.020 (1.016, 1.025)	Reference
Black	0.973 (0.942, 1.004)	0.002	1.052 (1.025, 1.08)	0.354	1.014 (0.998, 1.030)	0.771	1.019 (1.006, 1.032)	0.798
Other[b]	1.063 (1.016, 1.112)	0.148	1.009 (0.99, 1.027)	0.003	0.994 (0.975, 1.013)	0.030	0.981 (0.972, 0.991)	<0.001
Medicaid eligibility								
Ineligible	1.024 (1.012, 1.037)	0.683	1.039 (1.031, 1.046)	0.907	1.014 (1.006, 1.022)	0.521	1.019 (1.015, 1.024)	0.038
Ever eligible	1.031 (1.004, 1.059)		1.04 (1.026, 1.053)		1.019 (1.006, 1.032)		1.011 (1.004, 1.017)	

Region								
Midwest	1.127 (1.097, 1.158)	Reference	1.099 (1.067, 1.132)	Reference	1.075 (1.054, 1.096)	Reference	1.072 (1.053, 1.09)	Reference
Northeast	1.031 (1.011, 1.051)	<0.001	0.960 (0.940, 0.980)	<0.001	1.024 (1.012, 1.037)	<0.001	1.004 (0.993, 1.016)	<0.001
Southeast	0.964 (0.931, 0.998)	<0.001	1.073 (1.051, 1.096)	0.193	0.996 (0.977, 1.015)	<0.001	1.019 (1.005, 1.033)	<0.001
Southwest	1.044 (1.000, 1.089)	0.003	1.018 (0.986, 1.052)	0.001	0.946 (0.922, 0.971)	<0.001	1.066 (1.046, 1.086)	0.687
West	1.083 (1.050, 1.117)	0.057	1.016 (0.994, 1.039)	<0.001	1.013 (0.996, 1.030)	<0.001	0.982 (0.972, 0.993)	<0.001
Urbanization								
Urban	1.012 (0.999, 1.025)	<0.001	1.061 (1.051, 1.071)	<0.001	1.004 (0.996, 1.012)	0.001	1.033 (1.028, 1.039)	0.001
Rural	1.185 (1.028, 1.366)		1.085 (1.039, 1.134)		1.122 (1.048, 1.201)		1.045 (1.018, 1.073)	

[a]P-value for interaction term was estimated by the Wald test.

[b]Other included Asian, Hispanic, American Indian, or Alaskan Native, and unknown.

All results were presented in the unit of per IQR change of exposure. In the AD cohort, the IQRs for UFPs and $PM_{2.5}$ were 3701.6 particles/cm^3 and 1.9 μg/m^3, respectively. In the ADRD cohort, the IQRs for UFPs and PM2.5 were 3668.5 particles/cm^3 and 1.9 μg/m^3, respective

Table S3. Hazard ratios and 95% CIs in UFPs and $PM_{2.5}$ associated with AD or ADRD from single-pollutant and bi-pollutants models.

	AD	ADRD
**UFPs (particles /cm^3)**		
Single-pollutant	1.026 (1.014, 1.038)	1.016 (1.008, 1.023)
Bi-pollutants	1.016 (1.003, 1.028)	1.011 (1.003, 1.019)
**$PM_{2.5}$(μg/m^3)**		
Single-pollutant	1.039 (1.030, 1.045)	1.017 (1.012, 1.021)
Bi-pollutants	1.035 (1.027, 1.042)	1.015 (1.011, 1.020)

Table S4. Hazard ratios and 95% CIs of UFPs and $PM_{2.5}$ associated with AD or ADRD with a 5-year clean period.

**Exposure**	**AD**	**ADRD**
**UFPs (particles/cm^3)**		
Continuous[a]	1.027 (1.015, 1.039)	1.014 (1.006, 1.021)
Quartile 1	Reference	Reference
Quartile 2	1.015 (1.008, 1.022)	1.012 (1.008, 1.016)
Quartile 3	1.032 (1.014, 1.049)	1.033 (1.023, 1.044)
Quartile 4	1.039 (1.019, 1.058)	1.038 (1.027, 1.049)
**$PM_{2.5}$ (μg/m^3)**		
Continuous[a]	1.037 (1.030, 1.045)	1.016 (1.012, 1.020)
Quartile 1	Reference	Reference
Quartile 2	1.047 (1.032, 1.063)	1.038 (1.030, 1.047)
Quartile 3	1.078 (1.061, 1.096)	1.057 (1.047, 1.067)
Quartile 4	1.099 (1.079, 1.119)	1.059 (1.048, 1.071)

[a]Results were presented in the unit of per IQR change of exposure. In the AD cohort, the IQRs for UFPs and $PM_{2.5}$ were 3681.3 particles/cm^3 and 1.9 μg/m^3, respectively. In the ADRD cohort, the IQRs for UFPs and $PM_{2.5}$ were 3641.2 particles/cm^3 and 1.9 μg/m^3, respectively.

Table S5. Hazard ratios and 95% CIs of UFPs and $PM_{2.5}$ associated with AD or dementia among the non-movers.

Exposure	AD	ADRD
**UFPs (particles/cm^3)**		
Continuous[a]	1.033 (1.021, 1.046)	1.021 (1.014, 1.029)
Quartile 1	Reference	Reference
Quartile 2	1.032 (1.015, 1.050)	1.035 (1.025, 1.046)
Quartile 3	1.045 (1.026, 1.065)	1.042 (1.031, 1.053)
Quartile 4	1.057 (1.035, 1.080)	1.048 (1.034, 1.061)
**$PM_{2.5}$ (μg/m^3)**		
Continuous[a]	1.036 (1.028, 1.043)	1.015 (1.011, 1.019)
Quartile 1	Reference	Reference
Quartile 2	1.044 (1.028, 1.060)	1.038 (1.030, 1.047)
Quartile 3	1.069 (1.052, 1.086)	1.055 (1.045, 1.065)
Quartile 4	1.095 (1.075, 1.115)	1.060 (1.049, 1.071)

Abbreviations: AD, Alzheimer's disease; CI, confidence interval; IQR: interquartile range; $PM_{2.5}$, fine particulate matter; UFPs, ultrafine particles.
[a]Results were presented in the unit of per IQR change of exposure. In the AD cohort, the IQRs for UFPs and $PM_{2.5}$ were 3701.6 particles/cm^3 and 1.9 μg/m^3, respectively. In the dementia cohort, the IQRs for UFPs and $PM_{2.5}$ were 3668.5 particles/cm^3 and 1.9 μg/m^3, respectively.

Table S6. Hazard ratios and 95% CIs of per IQR increase in UFPs and $PM_{2.5}$ associated with AD or ADRD, additionally adjusting for comorbidities.

Outcome	Models	UFPs	$PM_{2.5}$
AD	Main model	1.026 (1.014, 1.038)	1.037 (1.030, 1.045)
	Main model+stroke	1.026 (1.014, 1.038)	1.035 (1.028, 1.042)
	Main model+hypertension	1.025 (1.013, 1.037)	1.034 (1.027, 1.041)
	Main model+depression	1.023 (1.011, 1.035)	1.038 (1.030, 1.045)
ADRD	Main model	1.016 (1.008, 1.023)	1.017 (1.012, 1.021)
	Main model+stroke	1.016 (1.009, 1.024)	1.013 (1.009, 1.017)
	Main model+hypertension	1.014 (1.007, 1.021)	1.011 (1.007, 1.015)
	Main model+depression	1.013 (1.005, 1.020)	1.017 (1.013, 1.021)

All hazard ratios were calculated using the same IQRs with the main analysis. In the AD cohort, the IQRs for UFPs and $PM_{2.5}$ were 3701.6 particles/cm^3 and 1.9 μg/m^3, respectively. In the ADRD cohort, the IQRs for UFPs and $PM_{2.5}$ were 3668.5 particles/cm^3 and 1.9 μg/m^3, respectively.

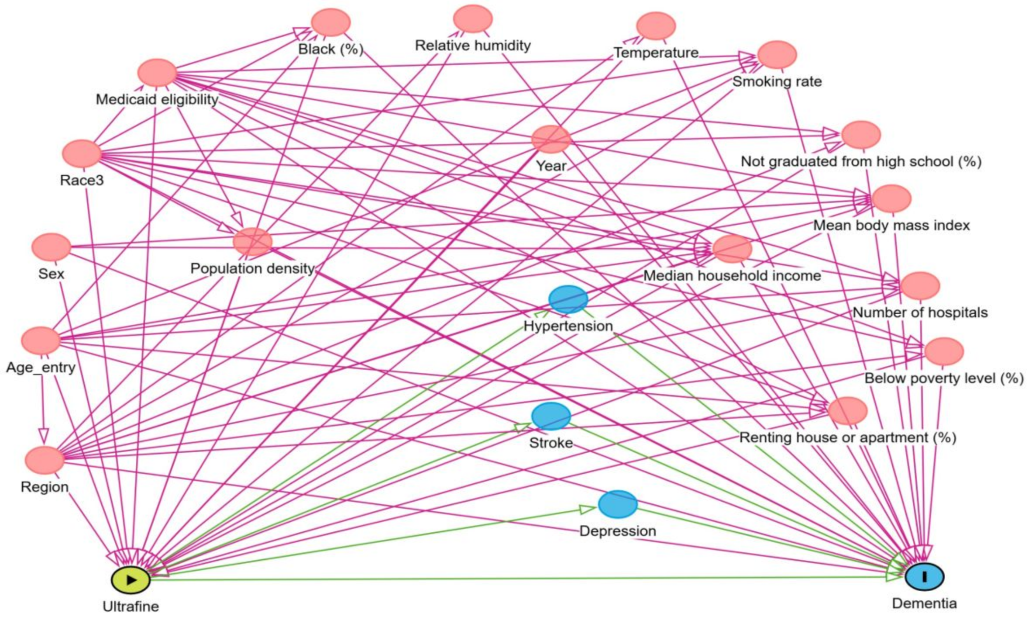

Figure S1 Directed acyclic graph for the associations of ultrafine and $PM_{2.5}$ with dementia and AD, created with the help of DAGitty.net (www.dagitty.net, accessed on February 8, 2025).

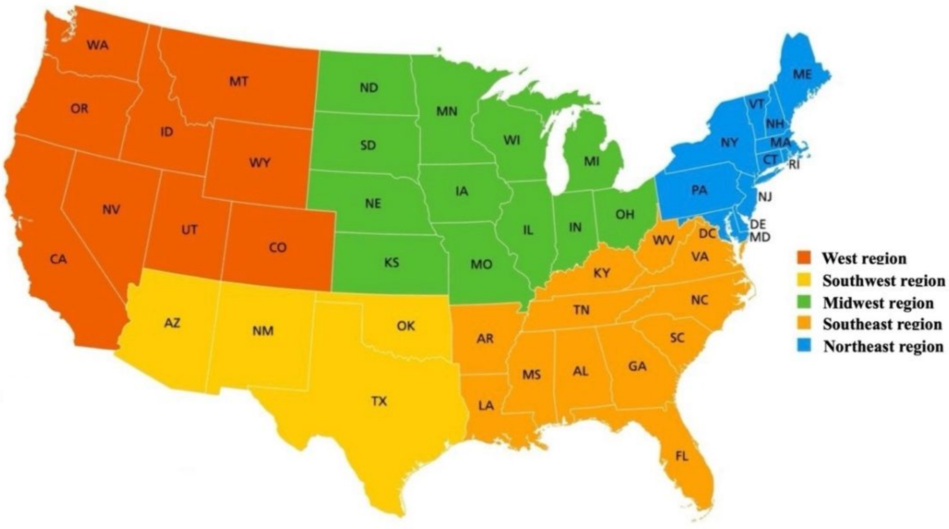

Figure S2 Map of geographical regions (West, Midwest, Southwest, Southeast, Northeast) in the contiguous United States.
